# Hyaluronan-Related Granulomatous Synovitis, Adipositis, and Osteomyelitis in the Osteoarthritic Knee: A Morphological Case Series of Three Patients

**DOI:** 10.3390/ijms26168073

**Published:** 2025-08-21

**Authors:** Vera Lyalina, Gulnara Eshmotova, Alexandra Karavan, Andrey Korshunov, Alexey Zarov, Anton Bonartsev, Natalia Serejnikova, Alexey Prizov, George Airapetov, Alexey Volkov

**Affiliations:** 1 School of Clinical Medicine Storozhakov Internal Diseases Department, Pirogov Russian National Research Medical University, 117513 Moscow, Russia; vera_lyalina@mail.ru; 2 St. Alexy Metropolitan of Moscow Central Hospital, 119071 Moscow, Russia; geshmotova@mail.ru (G.E.); dr.korshunov64@mail.ru (A.K.); zarow@mail.ru (A.Z.); i@bonelab.ru (A.V.); 3 Department of Pathological Anatomy, Medical Institute, Patrice Lumumba Peoples’ Friendship University of Russia (RUDN University), 117198 Moscow, Russia; karavanas2001@gmail.com; 4 Faculty of Biology, Lomonosov Moscow State University, 119234 Moscow, Russia; ant_bonar@mail.ru; 5 Institute for Regenerative Medicine, Sechenov First Moscow State Medical University (Sechenov University), 119991 Moscow, Russia; 6 Department of Traumatology and Orthopedics, Medical Institute, Patrice Lumumba Peoples’ Friendship University of Russia (RUDN University), 117198 Moscow, Russia; aprizov@yandex.ru (A.P.); airapetovga@yandex.ru (G.A.)

**Keywords:** intraarticular hyaluronan, osteoarthritis, viscosupplementation, pathomorphology, synovitis, adipositis, osteomyelitis, foreign body reaction

## Abstract

Intra-articular hyaluronan injections represent a widely used and generally safe therapeutic approach for knee osteoarthritis (OA). However, the side effects of this treatment remain insufficiently studied. Acute post-injection reactions, particularly those arising from an improper technique resulting in the deposition of the therapeutic agent into joint tissues, are well-documented. In contrast, chronic hyaluronan-induced inflammatory responses have received scant attention in the scientific literature. The aim of this study is to characterize for the first time the morphological patterns of chronic granulomatous inflammation induced by exogenous hyaluronan (e-HA) in osteoarthritic knees, focusing on three distinct tissue reactions: synovitis, adipositis, and osteomyelitis. Using a three-case series approach and morphological analysis, we identified e-HA penetration pathways; described associated foreign body responses in the synovial, adipose, and bone tissues of the joints; and emphasized the clinical relevance of these underreported adverse effects. These observations highlight an understudied phenomenon—an active conflict between e-HA and joint tissues that recognize it as a foreign body. The prevalence, clinical significance, and prognostic implications of this phenomenon require further investigation.

## 1. Introduction

Hyaluronic acid, also known as hyaluronan, is a glycosaminoglycan and a key component of the synovial environment. Hyaluronan is synthesized by the synovial membrane, imparting viscosity to the synovial fluid, which in turn ensures the gliding of articular surfaces and contributes to the nutrition of the articular cartilage [1]. With aging, a progressive decline in both the quantity (30–50% by age 70) and molecular weight (from >2 MDa to <0.5 MDa) of endogenous hyaluronan is observed [2,3], which is considered one of the key aspects in the pathogenesis of cartilage degeneration and OA (osteoarthritis) [4]. The intra-articular administration of standardized e-HA (exogenous hyaluronan) aims to restore the quantity and quality of this substance in the synovial environment. This therapeutic strategy has accordingly been designated as «viscosupplementation» [5,6]. e-HA exhibits chondroprotective and anti-inflammatory effects while also improving joint lubrication and shock absorption [7,8,9,10]. Despite ongoing debates regarding the efficacy of intra-articular e-HA injections, viscosupplementation is widely used in OA treatment and is endorsed by most clinical guidelines [6,11,12,13].

Intra-articular e-HA injections have been utilized in clinical practice for approximately 30 years [14], with e-HA preparations generally demonstrating a favorable safety profile [15]. However, this treatment modality is not free from adverse effects, which remain poorly understood to date [6]. Specifically, local adverse events occur in 3.7–8.5% of intra-articular e-HA injections [6,12]. Acute post-injection aseptic arthritis may develop within 3–4 h to 5–6 days after administration, typically persisting for several days [16,17], though in some cases lasting up to 3 weeks [18]. The inflammatory process manifests as pain, swelling, and restricted joint mobility. The severity varies considerably, with some cases progressing to pseudo-septic reactions or a severe acute local reaction (SALR) [16,17,19].

The mechanisms underlying these local reactions remain unclear and are likely heterogeneous [16]. Potential causal factors include immunological reactivity due to the sensitization of the synovial environment to components of e-HA. This has prompted an investigation into specific product characteristics, such as the presence or absence of cross-linking and animal-derived versus bacterial fermentation origins. However, recent analytical evidence demonstrates no significant differences in the adverse event risk between e-HA preparation types [17]. Another potential cause of acute local reactions is believed to be the inadvertent infiltration of e-HA into the synovial tissue or the Hoffa fat pad due to improper injection techniques [17,20,21]. In addition, it has been established that e-HA can induce not only acute but also chronic inflammatory process within the joint, with current evidence limited to isolated case reports [22,23,24,25].

We present the morphological case series of three patients who received multiple intra-articular injections of various e-HA preparations and underwent knee arthroplasty for end-stage severe OA. The objective of our research is to characterize the chronic granulomatous inflammation induced by e-HA in osteoarthritic knee joints, highlighting three distinct tissue-specific patterns: synovitis, adipositis, and osteomyelitis. By analyzing these cases, we aim to achieve the following:

1. Provide the first detailed morphological description of e-HA-triggered foreign body reactions across synovial, adipose, and osseous tissues.
2. Identify potential pathways of e-HA penetration into different joint compartments.
3. Emphasize the underreported conflict between e-HA and host tissues, which perceive it as a foreign material.

This study underscores the need for further research into the prevalence, clinical impact, and long-term consequences of chronic e-HA-induced inflammation.

## 2. Results

**Figure 1:**
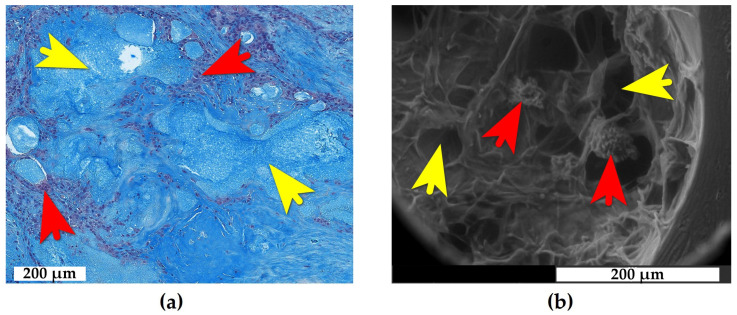
e-HA (exogenous hyaluronan) deposits in synovial and bone tissues. (**a**) Synovial membrane: e-HA inclusions surrounded by FBGCs, Alcian blue stain, brightfield microscopy; (**b**) Bone tissue: intraosseous honeycomb-like cavities with low electron density surrounded by FBGCs, environmental SEM (low vacuum, uncoated sample). Yellow arrows: e-HA-filled cavities. Red arrows: FBGCs.

In all patients and across all examined tissues, we identified inclusions of monomorphic, basophilic, and acellular material that exhibited specific staining for mucopolysaccharides. These deposits were consistently surrounded by foreign body giant cells (FBGCs) (Figure 1a). The scanning electron microscopy (SEM) of subchondral bone samples revealed extensive low-electron-density areas (similar in density to water) within yellow bone marrow. FBGCs were located peripherally along the margins of these cavities (Figure 1b). Hence, morphological and histochemical findings confirmed the deposits as e-HA.

### 2.1. Adipose Tissue Response to e-HA

**Figure 2:**
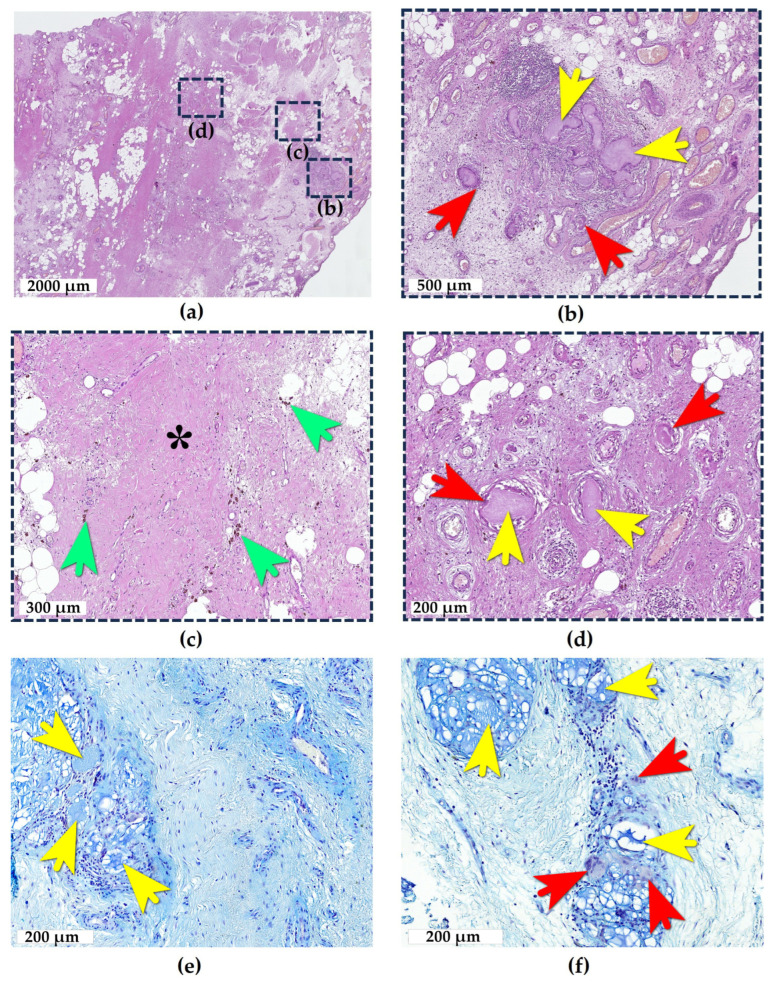
e-HA deposits in adipose tissue. (**a**) General view of adipose tissue showing edema and partial replacement by connective tissue. The interest zones (b), (c), and (d), indicated by squares with a dotted line, represent magnified fields of view of various areas in overview (**a**); (**b**) First e-HA bolus appearing as a focus of mucinous substance droplets surrounded by giant cell reaction, with intervening loose fibrous connective tissue. Note perivascular lymphoid infiltration in surrounding capillaries; (**c**) fibrosis and hemosiderin deposition at the periphery of e-HA boluses; and (**d**) second e-HA bolus composed of residual small droplets with areas of complete resorption (granulomas) alternating with dense fibrous connective tissue strands. (**e**) e-HA clusters stained with Alcian blue in adipose tissue; (**f**) phagocytosis of e-HA microdroplets stained with Alcian blue by FBGCs. Hematoxylin and Eosin stain (**a**–**d**), Alcian blue stain (**e**,**f**), and brightfield microscopy. Yellow arrows: e-HA-filled inclusions. Red arrows: FBGCs. Green arrows: hemosiderin deposits. Asterisk: dense fibrous connective tissue.

The adipose tissue of Hoffa’s fat pad (Figure 2a) exhibited edema, with the focal replacement by loose and/or dense fibrous connective tissue containing numerous blood vessels of varying caliber showing both arterial and venous differentiation, with predominant arterial congestion. The vascular walls demonstrated edema and dissection. Small- and medium-sized blood vessels exhibited neutrophil rolling, though no tissue neutrophilic infiltration was present. However, there was a diffuse focal infiltration of the adipose tissue by lymphocytes, macrophages, and FBGCs, indicating the development of moderate adipositis. The adjacent synovial membrane consisted of a single layer of synoviocytes without villous hyperplasia or hypertrophy.

In the lateral portion of the fat pad, two distinct e-HA boluses were identified. The first bolus was located 0.7 mm deep to the synovial lining and consisted of a rounded focus of aggregated multiple droplets surrounded by FBGCs (Figure 2a,b). Between the droplets, there was intervening loose fibrous connective tissue with prominent lymphocytic infiltration. At the periphery of the bolus, an emerging lymphoid follicle was noted. Adjacent capillaries showed perivascular lymphoid infiltration. The periphery of the bolus demonstrated the formation of dense fibrous connective tissue that was progressively replacing the area, along with a thin rim of a hemosiderin deposition indicative of prior tissue injury and hemorrhage (Figure 2c). These features characterized this as an injection bolus of e-HA surrounded by chronic granulomatous inflammation (with a duration exceeding 14 days). Additionally, the adipose tissue contained diffuse foci of a granulomatous inflammatory reaction surrounding individual droplets of the mucinous substance. These foci showed varying stages of development, evidenced by different degrees of e-HA resorption, with some demonstrating final stages of resorption and replacement by the fibrous tissue.

At a depth of 0.5 mm from the synovial lining and 3.5 mm from the first bolus, a second e-HA bolus was found, surrounded by dense connective tissue (Figure 2d). This bolus consisted of small residual droplets interspersed with areas of complete resorption appearing as granulomas. Between the residual droplets and granulomas, there were layers of dense fibrous connective tissue (scar formation).

Both large granulomatous inflammatory foci shown in Figure 2, along with smaller scattered droplets, were located in the lateral fat pad within an area of 6 × 6 mm^2^ (3.6 cm^2^)—corresponding to the standard anterolateral approach for intra-articular knee injections and at depths consistent with typical injection needle lengths. These findings clearly represent the sequelae of repeated inaccurate intra-articular e-HA injections of varying durations—that is, deposited preparation boluses that failed to reach the joint space and remained in adipose tissue at different stages of conflict (ranging from active granulomatous inflammation to post-inflammatory fibrotic changes).

### 2.2. Synovial Tissue Response to e-HA

**Figure 3:**
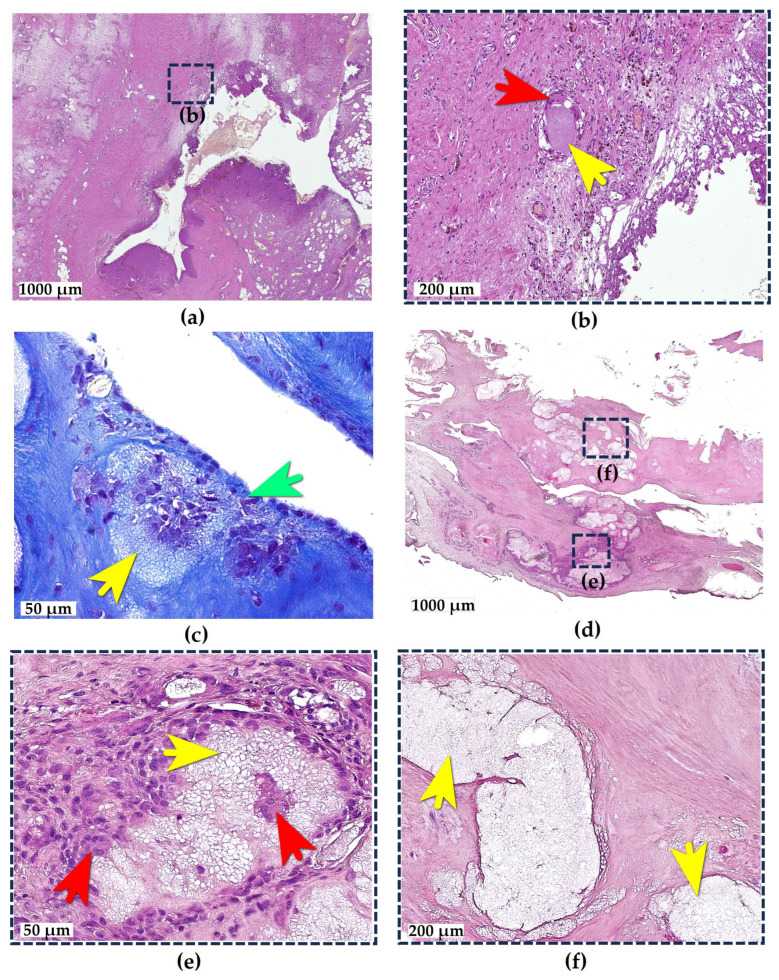
e-HA deposits in synovial tissue. (**a**) Areolar-type synovial membrane showing villous hyperplasia, edema, and scattered e-HA droplets. The interest zone (b), indicated by a square with a dotted line, represents a magnified field of the view of a part of the overview (**a**); (**b**) e-HA droplet surrounded by FBGCs within dense fibrous connective tissue of synovium; (**c**) phagocytosis of e-HA by synoviocytes; (**d**) fibrous-type synovial membrane with villous hypertrophy, fibrosis, and large e-HA boluses; (**e**) fresh e-HA deposit with prominent granulomatous reaction; and (**f**) long-standing e-HA focus “walled off” by dense fibrous connective tissue. The interest zones (e), and (f), indicated by squares with a dotted line, represent magnified fields of view of various areas in overview (**d**). Hematoxylin and Eosin stain (**a**,**b**,**d**–**f**), Mallory’s stain (**c**), brightfield microscopy. Yellow arrows: e-HA-filled inclusions. Red arrows: FBGCs. Green arrow: tissue phagocytosis site.

In samples of areolar-type synovial membranes, villous hyperplasia and focal pronounced synoviocyte proliferation were observed (Figure 3a). The subsynovial layer exhibited edema and hemosiderosis. The joint surface demonstrated erythrocytes and fibrin strands, with the adjacent tissue showing linear aggregates of hemosiderophages engulfing erythrocytes extravasated from congested vessels. The synovial tissue was infiltrated by lymphocytes, macrophages, and FBGCs. Additional findings included vasculitis, vascular fibrosis, lumen obliteration, and erythrocyte sludging. These morphological changes were consistent with chronic synovitis in OA.

Scattered small e-HA droplets with similar characteristics were identified in some specimens of all patients, with no compact bolus structures observed in any case (Figure 3b). As shown in the representative sample, the e-HA droplets were consistently located within the subsynovial layer at depths of 0.3–1 mm, spaced 2.5 mm, 1.6 mm, and 3 mm apart. These deposits were associated with granulomatous inflammation at varying stages. Active phase droplets were surrounded by connective tissue microcapsules containing numerous FBGCs. Late-stage droplets showed resorption and fibrous replacement.

The presence of droplets in synovial areas not corresponding to injection sites, along with their scattered distribution, suggests that e-HA entered the tissues through a pathway other than direct injection. Them beneath the surface location of droplets (within 0.3–1 mm of the synovial lining) indicates their likely penetration from the joint cavity into the synovium. This hypothesis is supported by our observation of the tissue phagocytosis process (e-HA droplets passing through the superficial synovial layer into deeper tissue), which we identified in one of the specimens (Figure 3c).

In the fibrous-type synovial membrane sample obtained from the suprapatellar pouch, a different pattern was observed. Two e-HA boluses were found within the tissue (Figure 3d). The compact intramural location of these e-HA fragments in the area corresponding to the superior injection approach suggests they resulted from needle displacement.

The boluses appeared to differ in their duration of presence. The more recent bolus showed an active granulomatous reaction at its periphery, with numerous macrophages and FBGCs actively resorbing e-HA (Figure 3e). The older bolus demonstrated large deposits of unresorbed e-HA without a granulomatous reaction but with the significant fibrosis of the surrounding synovial tissue (Figure 3f). This suggests that, unlike the final stages of granulomatous reactions observed in the adipose tissue and areolar synovium, the fibrous synovial membrane tends to encapsulate rather than completely resorb e-HA deposits.

### 2.3. Bone Tissue Response to e-HA

**Figure 4:**
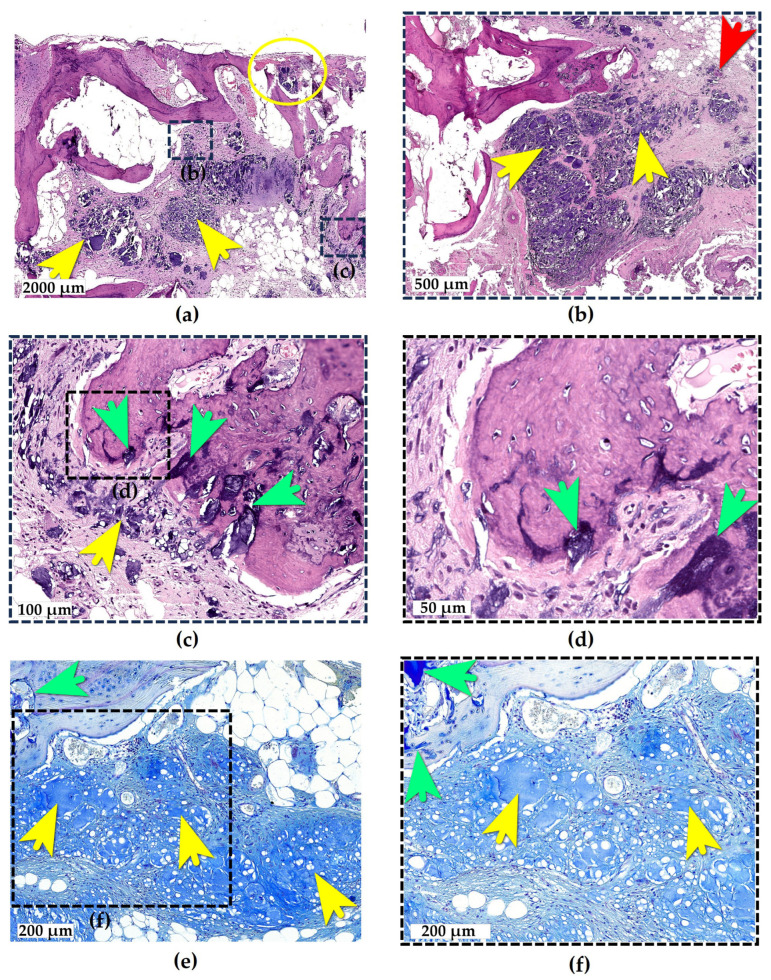
e-HA deposits in bone tissue. (**a**) The overall architecture of the damaged subchondral bone. Large e-HA foci are visible in the bone marrow. The interest zones (b), and (c), indicated by squares with a dotted line, represent magnified fields of view of various areas in overview (**a**); (**b**) section of an e-HA bolus in bone marrow, composed of e-HA droplets, FBGCs, and strands of connective tissue at varying maturation stages; (**c**) reparative osteogenesis at sites adjacent to the e-HA bolus; and (**d**) phagocytosis of e-HA microdroplets by bone cells and their incorporation into the reticulo-fibrous bone tissue formed during reparative osteogenesis. The interest zone (d), indicated by a square with a dotted line, represents a magnified field of the view of a part of the overview (**c**). (**e**) e-HA clusters stained with Alcian blue in bone and reticulo-fibrous bone tissue; and (**f**) phagocytosis of e-HA microdroplets stained with Alcian blue by bone cells and their incorporation into the reticulo-fibrous bone tissue. The interest zone (f), indicated by a square with a dotted line, represents a magnified field of the view of a part of the overview (**e**). Hematoxylin and Eosin stain (**a**–**d**), Alcian blue stain (**e**,**f**), and brightfield microscopy. Yellow circle: the surface localization of the recent e-HA deposit. Yellow arrows: e-HA-filled inclusions. Red arrow: FBGCs. Green arrows: sites of tissue phagocytosis.

e-HA deposits were identified in a bone fragment obtained from the weight-bearing articular surface of the medial tibial condyle (Figure 4a). The examined fragment consisted of subchondral bone with a complete loss of the hyaline cartilage coverage. The bone tissue exhibited osteosclerosis, disorganization, and the uneven thickness of the trabeculae, along with multiple areas of structural disruption and rarefaction. These morphological changes were consistent with end-stage OA.

A small focus of e-HA was observed on the surface of the destructively altered subchondral bone (Figure 4a, yellow circle), bordered superficially by a fresh blood clot. The e-HA microdroplet was surrounded by a giant cell reaction, with no evidence of connective tissue formation around it, indicating its relatively recent deposition (no more than 10–14 days old). This most likely represents an e-HA droplet that became fixed to the bone surface sometime after the intra-articular injection.

In the deeper bone tissue layers, a large e-HA deposit (3.2 × 3 mm) was identified, composed of several closely aggregated foci, likely representing fragments of an originally unified injection bolus (Figure 4a, yellow arrows). The penetration of this deposit into the deeper tissue presumably occurred through cracks in the exposed subchondral bone.

The bolus fragments of e-HA alternated with FBGCs and areas of connective tissue at varying stages of maturity (Figure 4b). The presence of a well-formed fibrous capsule surrounding the bolus indicates it had been present in the tissue for at least one month.

Foci of reparative osteogenesis were observed at the periphery of the deposit (Figure 4c,d). e-HA microdroplets measuring 15–20 μm in diameter, upon contacting the osteoid, became incorporated into its structure and gradually disappeared at sites of direct contact with osteocytes.

Consequently, the e-HA bolus in the subchondral bone induced granulomatous inflammation resembling aseptic osteomyelitis, characterized by the encapsulation of the deposit and its gradual resorption.

## 3. Discussion

Granulomatous reactions to e-HA are well-documented across all clinical applications [26,27,28]. The most extensively studied complications are dermatological, primarily due to the superficial location of clinically apparent inflammatory processes and the relative ease of obtaining targeted skin or subcutaneous tissue biopsies [29,30,31,32,33]. In contrast, the morphological verification of joint inflammation presents significant challenges, as it requires either deep, controlled cavity biopsies or the examination of substantial surgical specimens—procedures not routinely performed in clinical practice. Reports of granulomatous reactions associated with intra-articular e-HA injections emerged during the initial years of this treatment’s widespread adoption [22,23]. However, at the time, the phenomenon did not receive sufficient research attention—perhaps partly due to the prevailing perception of its exceptional rarity as a complication [34]. To date, publications on e-HA-associated articular granulomatosis remain scarce, though they continue to appear intermittently [35]. Such adverse reactions related to e-HA were found not only in clinical practice but also in animal studies. Thus, after the injection of Hylan G-F 20 into the rat knees, foreign bodies surrounded by macrophages were observed in the synovium at Hoffa’s fat pad [36]. While existing reports confirm the occurrence of such granulomatous processes, they fail to provide comprehensive explanations for how e-HA penetrates into the examined tissues, particularly in cases where injection technique errors can be ruled out.

When discussing inadvertent intra-articular injections in the knee, Hoffa’s fat pad represents the most likely site for potential granuloma formation. This predisposition stems from the technical specifics of the most commonly used anterolateral approach for intra-articular injections [37,38]. Early studies (later confirmed by ultrasound-guided investigations) demonstrated that without imaging guidance, the frequency of ectopic injections may approach 30% [39,40]. In such cases, the needle may indeed fail to enter the joint cavity, resulting in the deposition of the e-HA preparation within the fat pad. This complication is well-recognized in clinical practice and is considered one of the causes of post-injection local reactions [17,21]. However, the proportion of such complications among all post-injection reactions remains unknown, and the morphological characteristics of the resulting inflammation have not been previously described. Our observations demonstrate the development of not only acute but also chronic granulomatous inflammation in the fat pad surrounding the e-HA bolus, with subsequent fibrosis formation—a process that likely occurs after each inadvertent injection.

Another frequently employed approach for intra-articular knee injections is the lateral patellar (or superolateral) approach [41,42]. The use of this approach may also result in an improper needle placement and e-HA infiltration into the synovial membrane [41]. In our examination of synovial specimens from this region, we identified two e-HA boluses that provoked different tissue responses: one exhibiting granulomatous inflammation with active resorption, while the other showed encapsulation without resorption. These findings document a previously uncharacterized mode of interaction between synovial tissue and e-HA. While this isolated observation does not permit definitive conclusions regarding whether the encapsulation process is tissue-specific, dependent on particular e-HA preparation properties, or influenced by the administered volume, it nevertheless provides evidence for the existence of diverse e-HA-associated tissue responses.

An inadvertent injection represents a well-known and relatively common complication of intra-articular injections in general and as such constitutes an expected pathway for e-HA entry into joint tissues. However, the present findings demonstrate that e-HA may also reach joint tissues through mechanisms other than the direct injection delivery. We documented the phenomenon of active phagocytosis of e-HA droplets from the joint cavity by the synovial tissue. The phagocytosis of foreign material represents one of the fundamental properties of synovial tissue that mediates its protective cleansing function [43]. This process is known to facilitate foreign body reactions to various particulate matter, including metal, polyethylene, and cement particles generated during arthroplasty procedures [44,45,46]. Similarly, the phagocytosis of free fragments of the degenerated articular cartilage is well-established and considered an important contributor to chronic synovial inflammation in OA [43,47,48,49]. The obtained data do not permit conclusions regarding either the timing of the synovial phagocytosis initiation following the e-HA administration or its potential dependence on the quantity or specific characteristics of e-HA preparations. However, they clearly demonstrate that even when properly injected into the joint space, e-HA is recognized by the synovial environment as a foreign substance. Moreover, the synovium appears to actively eliminate it through phagocytic clearance.

Bone tissue may similarly become involved in pathological interactions with e-HA. Reports of various bone complications associated with e-HA are limited to isolated publications that fail to specify the pathways by which e-HA penetrates bone tissue [35,50]. As for osteomyelitis specifically, there is only one case report in the literature about mandibular osteomyelitis after a hyaluronic acid injection [51]. Our observations demonstrate that in cases of articular cartilage loss, e-HA can passively infiltrate into deep layers of subchondral bone. This penetration leads to the development of granulomatous osteomyelitis and osseous phagocytosis of the foreign material by osteocytes. Consequently, it appears appropriate to reconsider the rationale for intra-articular e-HA injections in patients with late-stage OA—not merely due to the absence of cartilage as a target for lubrication and chondroprotection, but more importantly because of the risk of iatrogenic osteomyelitis, a currently unrecognized complication.

Collectively, our findings provide objective evidence of a foreign body conflict occurring in all articular tissues that come into passive or active contact with e-HA. In contrast to its well-established anti-inflammatory properties, these observations reveal a pro-inflammatory effect of e-HA. Within the current understanding of OA as a chronic and likely heterogeneous inflammatory process [52,53], the potential role of intra-articular e-HA injections as a cause of chronic iatrogenic inflammation emerges as a particularly significant concern. Acute local reactions following intra-articular e-HA injections are well-recognized in clinical practice. While the exact pathogenesis of these reactions remains incompletely understood, their incidence rates have been well documented, their clinical manifestations have been thoroughly characterized, and treatment protocols have been established [17]. In contrast, knowledge about chronic inflammatory processes induced by e-HA injections remains extremely limited, and their clinical significance and long-term consequences represent important areas for future investigation. This study highlights the complexity and ambiguity of interactions between articular tissues and therapeutic agents intended to restore normal joint physiology. Within the current trend toward the expanded use of injection therapies for OA [6], greater attention must be devoted to investigating potential negative effects of such treatments.

Our research has several limitations. This study was based exclusively on a morphological examination of randomly selected surgical specimens, and we lacked detailed clinical data about the patients, including information on the duration and frequency of intra-articular e-HA injections or the specific preparations used. Furthermore, our investigation included only three cases.

## 4. Materials and Methods

### 4.1. Patients

Surgical specimens were retrospectively analyzed from three randomly selected patients who underwent knee arthroplasty for end-stage severe OA. All patients presented with persistent chronic pain syndrome, significant functional impairment, and failure of conservative treatment. For several years prior to surgery, all patients had received multiple intra-articular injections of various (unspecified) e-HA preparations (Table 1).

Clinical data collection was intentionally restricted to core parameters such as essential demographics, basic clinical symptoms, and history of multiple hyaluronan injections, as this study primarily focused on comprehensive histopathological characterization rather than clinical correlations. Specific hyaluronan formulations were not analyzed due to (1) insufficient sample size for statistically valid inter-product comparisons (*n* = 3) and (2) this study’s principal focus on the evaluation of universal histopathological patterns rather than product-specific effects.

### 4.2. Technique of Surgery and Sampling of Histological Material

Tissue specimens were obtained following standard surgical protocols for knee arthroplasty. No targeted biopsy procedures were performed.

In all three cases, synovial membrane samples were available for examination. From each patient, areolar-type synovial tissue was collected from the capsulomeniscal junction region at the area of the medial and lateral meniscal bodies. A total of six synovial samples measuring 1 × 1–1.5 cm were analyzed. Additionally, one patient provided a fibrous-type synovial membrane specimen (1.2 × 2.5 cm) from the suprapatellar pouch of the knee joint.

In one case, a subtotally resected Hoffa’s fat pad (sample size 3 × 4 cm) was obtained for analysis.

Another case included a tibial plateau resection specimen with the medial compartment of 4 mm height and lateral compartment of 8 mm height. The plateau was sectioned transversely at 0.5 cm intervals. From these sections, the lateral and medial compartments as well as the intercondylar eminence region were examined.

### 4.3. Morphological Analysis

#### 4.3.1. Light Microscopy

All tissue samples were fixed in 10% neutral buffered formalin (Biovitrum, Saint Petersburg, Russia). The tibial plateau sections were decalcified in SoftDec solution (Biovitrum, Russia). Subsequently, the specimens were dehydrated in isopropanol using an automated tissue processor (Epredia STP120, Thermo Fisher Scientific, Waltham, MA, USA) and embedded in paraffin using a HistoStar tissue embedding station (Thermo Fisher Scientific, Waltham, MA, USA). Histological sections (4 μm thick) were prepared using a Leica RM 2125 RTS microtome (Leica Microsystems, Wetzlar, Germany) and stained with Hematoxylin and Eosin, Mallory’s trichrome, and Alcian blue (pH 2.5) (Biovitrum, Saint Petersburg, Russia). All staining procedures followed standard histological protocols [54,55]. All histological sections were examined under standardized light microscopy conditions (×50–400 magnification). Digital imaging of the slides was performed using a Hamamatsu NanoZoomer S20MD high-resolution slide scanner (Hamamatsu, Shizuoka, Japan) for detailed morphological assessment.

#### 4.3.2. Scanning Electron Microscopy (SEM)

To confirm the ultrastructural characteristics of basophilic deposits, SEM analysis was conducted on native tibial plateau specimens from Patient C. The samples were fixed in 10% neutral buffered formalin (Biovitrum, Saint Petersburg, Russia), rinsed in distilled water to remove residual fixative, and imaged under low-vacuum specific conditions (60–150 Pa pressure mode; 5–15 kV accelerating voltage; 5–10 mm working distance) using a Thermo Fisher Scientific Quattro S field-emission SEM (Thermo Fisher Scientific, Waltham, MA, USA) equipped with a large-field gaseous secondary electron detector for optimal surface topography imaging. This approach [56] allowed for high-resolution visualization of hyaluronan deposits while preserving their native morphology and minimizing artifacts associated with conventional SEM preparation.

## 5. Conclusions

We present the morphological description of chronic e-HA-induced granulomatous inflammation in joint tissues of patients with late-stage knee OA. Our findings demonstrate the different pathways through which e-HA penetrates articular tissues, including direct injection deposition, active tissue phagocytosis, and passive diffusion processes. This work provides the first detailed morphological characterization of the complete progression patterns of granulomatous adipositis and synovitis, along with the description of aseptic e-HA-induced osteomyelitis. Furthermore, we document the concurrent development of e-HA-induced granulomatosis affecting different tissues within the same joint. The obtained data underscore the importance of a more comprehensive investigation into the adverse effects of intra-articular treatments.

## Figures and Tables

**Table 1:** Characteristics of patients with OA (osteoarthritis) subjected to intra-articular e-HA (exogenous hyaluronan) injections.

Patients	Tissue Samples	e-HA-Induced Pathology
D., 73 y.o., female	Areolar synovial membrane	Synovitis
O., 58 y.o., male	Areolar synovial membrane and Hoffa fat pad	Synovitis and adipositis
S., 59 y.o., female	Areolar and fibrous synovial membrane and subchondral bone	Synovitis and osteomyelitis

## Data Availability

The data presented in this study are available upon request from the corresponding author.

## Abbreviations

The following abbreviations are used in this manuscript:

e-HA	Exogenous hyaluronan
OA	Osteoarthritis
SEM	Scanning electron microscopy
FBGCs	Foreign body giant cells
